# Practical Application of Aztreonam-Avibactam as a Treatment Strategy for Ambler Class B Metallo-β-Lactamase Producing Enterobacteriaceae

**DOI:** 10.3390/antibiotics13080766

**Published:** 2024-08-14

**Authors:** Darren W. Wong

**Affiliations:** Division of Infectious Diseases, University of Pittsburgh Medical Center, Pittsburgh, PA 15213, USA; wongdw@upmc.edu

**Keywords:** aztreonam-avibactam, MBL, NDM, ceftazidime-avibactam

## Abstract

Carbapenem-resistant Enterobacteriaceae infections are a considerable challenge for clinicians. In recent years, novel antibiotic options have resulted in a tremendous advance in medical therapy; however, current treatment options are primarily effective for resistance derived from serine-based carbapenemases. The Ambler class B metallo-β-lactamases (MBLs) remain a critical challenge with decidedly fewer effective options. One intriguing option for these MBL pathogens is the combination of ceftazidime-avibactam with aztreonam. While clinical experience with this regimen is limited, in vitro studies are promising, and limited case reports describe success with this regimen; however, significant challenges preclude widespread adoption of this novel treatment regimen. A systemic literature review was performed to offer recommendations based on current evidence for a practical strategy on how to best integrate the use of aztreonam with avibactam combination therapy.

## 1. Introduction

Carbapenemase-producing Enterobacteriaceae (CRE) are recognized as one of our most critical global healthcare threats. Patients with infection due to CRE have reported rates of 30-day mortality ranging from 24–44% as compared to 9–13% in patients with carbapenem-sensitive pathogens [1,2,3,4]. Resistance to carbapenems is most commonly mediated by carbapenem hydrolyzing β-lactamases, which account for 35–59% of CRE in the United States [5]. The Ambler Classification divides β-lactamases into four molecular classes, A–D, with classes A, C, and D utilizing a serine moiety and class B utilizing a metalloenzymatic zinc ion at its active site. Historically, CRE were treated with antimicrobials that had either high toxicity or suboptimal efficacy. This has incentivized drug development and resulted in the approval of several novel therapeutics that have significantly changed our treatment paradigm for CRE. Ceftazidime-avibactam, imipenem-cilastatin-relebactam, and meropenem-vaborbactam have all shown high efficacy for treatment of CRE infections, allowing for their adoption as the preferred option for susceptible carbapenemase producing pathogens [5,6]. However, a major limitation is that these novel inhibitor agents only restore bactericidal activity for the treatment of pathogens with serine-based carbapenemases, specifically the Ambler class A *Klebsiella pneumoniae* carbapenemase (KPC), and lack activity for Ambler class B metallo-β-lactamases (MBLs). While several bacterial species, specifically *Stenotrophomonas maltophilia*, possess inherent chromosomal carbapenamases, the most significant class B enzymes are the acquired Verona integron-encoded metallo-β-lactamases (VIMs), New Delhi metallo-β-lactamases (NDMs), and Imipenemase (IMP). Acquired VIM and IMP enzymes are most commonly associated with integrons or transposons, while NDMs are most commonly transmitted by plasmids harboring multiple resistance elements [7]. Due to their wide distribution and potential for proliferation, strategies to optimally treat pathogens possessing Ambler class B metallo-β-lactamases remain an unaddressed and critical need.

The widespread proliferation of Ambler Class B metalloenzymatic β-lactamases is a major concern. Infections due to NDM carbapenemase-producing bacteria have been described on all continents except Antarctica [8]. However, the burden of acquired, plasmid-mediated NDM-type MBLs is highest in South and Southeast Asia, with a particularly high prevalence in India, Bangladesh, and Pakistan [7]. Surveillance studies have identified NDM-harboring Enterobacteriaceae in samples of public tap water in India, and additional epidemiologic surveillance reports have raised concern due to rising prevalence rates in China and within the Middle East, where NDM MBLs now comprise the second most prevalent carbapenemase [7]. Consequently, while there is a need for novel therapeutic options for MBL CRE, the use of a combination of aztreonam and avibactam offers the possibility to reinvigorate and expand the spectrum of two current existing agents. The use of this combination has been studied in experimental models and small case series. However, a systematic review of this regimen’s efficacy and its potential clinical suitability and limitations has not been directly described.

## 2. Methods

A systematic review of the literature was performed to examine reports of treatment for metallo-β-lactamase-producing Gram-negative infections. A search was performed on 1/2/24 to identify any published literature regarding in vitro analysis of therapies as well as clinical management, with a focus on recent literature and novel therapeutics. Search parameters were utilized to screen for relevant references. Search terms “MBL” AND “CRE” using the PubMed search engine were performed. A second search on the PubMed search database was conducted with the search term “aztreonam-avibactam”, and all results were reviewed for relevance, with a preference for manuscripts with a recency in the last 2 years. Review articles were identified and briefly reviewed; however, priority was also given to manuscripts with primary research findings. References from identified articles were also reviewed to identify other relevant studies. The literature search was in accordance with current PRISMA guidance for systemic review.

## 3. Systemic Review Findings

**Figure 1:**
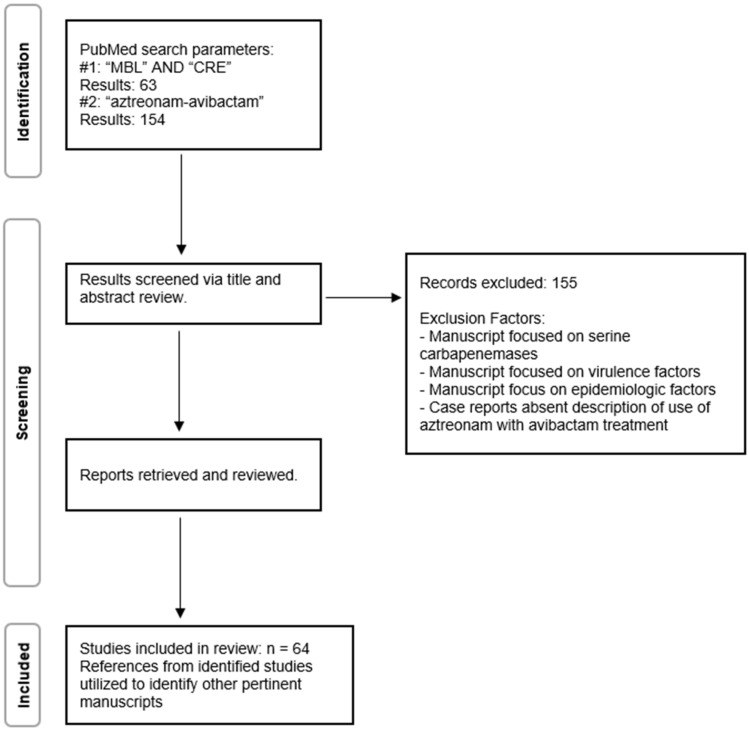
Literature Review Methodology.

A total of 63 manuscripts were identified following an initial “CRE” PubMed search. An additional 154 articles resulted from the “aztreonam-avibactam” search. These results were reviewed to identify relevant studies pertinent to the focused review. Manuscripts that primarily described virulence factors or epidemiologic factors were excluded, as they were deemed not to be the focus of this review. Additionally, single-case reports were excluded unless they described successful management utilizing an aztreonam and avibactam combination treatment regimen. There were a sizeable number of manuscripts that described the treatment of serine-based carbapenemase Enterobacteriaceae and were subsequently excluded. A total of 64 articles were determined to be relevant to analysis, which were used to identify additional relevant studies. The review methodology is graphically represented in Figure 1.

### 3.1. In Vitro Studies

The combination of aztreonam-avibactam has been shown to have high rates of in vitro sensitivity for CRE. Restored sensitivity to aztreonam following the addition of avibactam appears to be well described across multiple geographic regions and diverse microbial samples. The SENTRY Antimicrobial Surveillance Program collected 24,924 consecutive isolates of CRE between 2019 and 2021 from 69 medical centers in 36 countries within Europe, Asia, and Latin America. Amongst this diverse sample, the combination of aztreonam-avibactam inhibited 99.6% of all CRE, with activity consistent across geographical regions [9]. A surveillance study identified 1192 CRE isolates obtained from 33 hospitals within 5 countries of the Arabian Peninsula. Within this subset, OXA (54%) and NDM (42%) β-lactamases were common with the occurrence of dual carbapenemase production (12.8%), and interestingly, a notable subset of isolates were identified as absent carbapenemase production (12.7%). Despite these limitations, the aztreonam-avibactam susceptibility rate remained high (95.5%), with the caveat that the rate of non-susceptibility of aztreonam-avibactam reached 14.6% in the subset of CRE that were non-carbapenemase producers [10]. A multi-center study from China including 161 MBL-Enterobacteriaceae isolates found that avibactam reduced the minimum inhibitory concentration (MIC) of aztreonam-resistant isolates by more than 8-fold, with nearly 97% (156/161) of isolates inhibited by the combination of aztreonam-avibactam at ≤1 μg/mL [11]. Within the United States, between this same period, 2019 and 2021, 27,834 Enterobacteriaceae isolates were collected from 74 medical centers. While CRE rates were very low, 0.8%, 0.9%, and 1.1% of isolates in 2019, 2020, and 2021, respectively, aztreonam-avibactam inhibited > 99.9% of isolates, with only 3 isolates exhibiting an MIC > 8 μg/mL. Amongst the CRE isolates, 260 of 261 isolates were inhibited by aztreonam-avibactam with an MIC of ≤8 μg/mL [12]. In another study, Huang et. al. [13] collected 195 isolates of carbapenemase-producing CRE, comprising 143 MBL, 38 KPC, and 14 OXA-48 strains. Using broth microdilution, susceptibility to aztreonam-avibactam for each carbapenemase subtype was 96%, 100%, and 100%, respectively. An important additional finding was that NDM and VIM-producing *Escherichia coli* exhibited a lower susceptibility to aztreonam-avibactam at 77% and 75%, respectively, compared to other MBL-producing Enterobacteriaceae species, which were 100% susceptible. Bhatnagar et al. [14] described the results from 64 isolates acquired in the United States from 24 different states that were submitted to four regional Antibiotic Resistance Laboratory Network sites. These isolates were either PCR positive (confirmed by PCR testing) or non-susceptible to all β-lactams, including at least one of ceftazidime-avibactam or meropenem-vaborbactam. Amongst these 64 clinical isolates, the MIC50 and MIC90 values of aztreonam-avibactam were 0.5/4 μg/mL and 8/4 μg/mL, respectively. The addition of avibactam reduced the minimum inhibitory concentration (MIC) of aztreonam by a minimum of 4-fold in all isolates with a median reduction of 128-fold; most importantly, the combination restored susceptibility to aztreonam in 85% (51/60) of resistant isolates. A study by Vázquez-Ucha et al. [15] found that aztreonam-avibactam was highly active for a highly resistant subset of 55 clinical MBL-producing Enterobacteriaceae isolates obtained from a multi-center survey of 24 hospitals in Spain with an MIC ≤ 1 μg/mL for 92.7% of isolates. Similarly, Chen et al. [16] reported a large cohort of 1202 Enterobacteriaceae from clinical isolates comprising 10 species and acquired from 26 hospitals throughout 7 regions of China. Within this cohort, 119 CRE isolates were identified, with carbapenemase production detected in 87 isolates. Amongst this subgroup, aztreonam-avibactam exhibited sensitivity for 92% (110/119). Notably, amongst 77 Morganella isolates, the combination of aztreonam-avibactam susceptibility was only 84% (65/77), despite many of these isolates lacking carbapenemase production. An additional interesting finding was that MIC values for aztreonam-avibactam and ceftazidime-avibactam exhibited poor consistency. There were 43 clinical isolates that exhibited resistance > 16/4 µg/mL to ceftazidime-avibactam with a corresponding aztreonam-avibactam MIC ≤ 1/4 µg/mL, whereas 35 isolates exhibited elevated aztreonam-avibactam MIC with a lower MIC to ceftazidime-avibactam. In France, between 2012 and 2013, six regional hospitals collected 139 carbapenem-resistant Enterobacteriaceae clinical samples. Despite carbapenemase production being detected in only 2/139 isolates, all isolates exhibited aztreonam-avibactam MIC ≤ 4 µg/mL, with most cases < 1 µg/mL. Notably, while the majority of isolates exhibited carbapenem resistance due to outer membrane porin deficiencies in association with AmpC β-lactamase or extended-spectrum-β-lactamase (ESβL), this did not appear to impair aztreonam-avibactam penetration into the periplasmic space [17]. Emeraud et al. [18] reported results from 50 MBL-producing Enterobacteriacae, 3 MBL-producing *Pseudomonas aeruginosa*, and 5 multi-drug-resistant *Stenotrophomonas maltophilia* isolates. Susceptibility to the combination of ceftazidime-avibactam with aztreonam was determined using E-test strip superposition. Susceptibility to aztreonam was restored in 86% (43/50) of Enterobacteriaceae and all (5/5) Stenotrophomonas isolates. However, while aztreonam with ceftazidime-avibactam was deemed susceptible in 2 of 3 Pseudomonas isolates, the reduction in MIC was modest, at most 2-fold. Similar findings by Feng et al. [19] reported findings of 19 NDM-1-producing bacterial isolates collected in China, and the combination of ceftazidime-avibactam with aztreonam resulted in synergism and a 100% predicted response. A unique study by Terrier et al. [20] compared the in vitro activity of aztreonam-avibactam compared to cefiderocol in a diverse sample of 64 representative MBL-producing Enterobacteriaceae. In this study, aztreonam-avibactam susceptibility was 70.3%, an in vitro susceptibility lower than reported by other in vitro studies; however, it was still significantly higher than the identified cefiderocol susceptibility of 39.1%.

### 3.2. In Vitro Susceptibility Testing

One challenge with wider utilization of aztreonam-avibactam in clinical practice is the lack of a current co-formulation of these agents; thus, in vitro sensitivity testing has not been widely available or is not timely due to a need to send clinical specimens to reference laboratories. While microdilution techniques remain the reference standard, this technique is both resource intensive and not available for most clinical laboratories. Attempts to confirm in vitro susceptibility to aztreonam-avibactam have relied on several different methodologies. The most widely described testing method involves disc stacking (DS), which has been reported to have a high correlation to broth microdilution techniques [21]; however, strip stacking (SS), strip crossing (SX), and broth disk elution (DE) have also been used [22]. Lima et al. [23] reported success with a modified disk stacking method applying a ceftazidime-avibactam disk to an uninoculated agar surface that was then incubated for 2 h. After incubation, the disk was removed, and a bacterial suspension was inoculated onto the agar surface using a swab with an aztreonam disk, then applied in the precise location as the former removed the ceftazidime-avibactam disk. Using this method, a strong correlation was found between a disk inhibition zone diameter of ≥23 mm and a broth microdilution for aztreonam-avibactam of ≤1 μg/mL. Khan et al. [22] compared the performance characteristics of different methods to the broth microdilution reference standard for 16 representative carbapenemase-producing bacterial isolates encompassing 8 Enterobacteriaceae and 8 *Pseudomonas aeruginosa* strains. Khan and colleagues [22] found that the most accurate, precise, and reproducible methods were disc elution and both strip methods when utilizing MIC-test strips (DE, SS, SX), which were deemed to have categorical agreement with a reference standard with 100% sensitivity and specificity. This was followed closely by strip crossing with an E-test strip and strip stacking with E-test strips with 95% and 87.5% sensitivity, respectively. Disc stacking (DS), the most widely used technique clinically, had the lowest performance with 43%, although errors were mostly due to the incorrect classification of synergy-positive (sensitive) aztreonam-ceftazidime-avibactam strains as resistant. As these methods all represent non-standardized methodologies, Kelley et al. [24] investigated the correlation between broth microdilution and agar microdilution. Both agar and broth microdilutions resulted in 97% essential agreement within a 2-fold minimum inhibitory concentration (MIC) across a variety of conditions. However, high inoculum density (5 × 10^7^ CFU/mL) as opposed to standard inoculum (5 × 10^5^ CFU/mL) and low media pH (5.0) did result in a decrease in aztreonam-avibactam activity.

A recent addition is the approval of a single diffusion gradient aztreonam-avibactam test strip (developed by the Italian company Liofilchem). Deschamps et al. [25] screened 145 clinical isolates for sensitivity to aztreonam-avibactam with an E-test strip overlay, the Liofilchem diffusion gradient strip, and broth microdilution. All tested isolates produced at least one MBL and were resistant to aztreonam. With broth microdilution used as the reference standard, agreement in MIC occurred in strip overlay and diffusion gradient strip at a rate of 77% and 92%, respectively. Reassuringly, major errors were rare, occurring in strip overlay and diffusion gradient at respective rates of 1.4% and 0%. Similarly, Emilie et al. [26] reported 100% categorical agreement of the Liofilchem test strip with broth microdilution for 41 MBL-producing Enterobacteriacae strains.

### 3.3. Limitations of Aztreonam-Avibactam Therapy

The combination of aztreonam-avibactam does have some recognized limitations [27]. Mushtaq et al. [28] used broth microdilution to measure the in vitro susceptibility of both ceftazidime-avibactam and aztreonam-avibactam for 51 strains of Enterobacteriacae that exhibited carbapenemase-independent carbapenem resistance. All isolates were negative for detection of carbapenemases by PCR or the modified carbapenem inactivation method. In these isolates, the MIC of aztreonam-avibactam exceeded the susceptibility breakpoint in 10/51 isolates, comprising 7/20 *E. coli* and 3/12 *Enterobacter cloacae* strains. This contrasts with ceftazidime-avibactam, which retained activity for all but 2 isolates of *E. coli* and remained within the susceptible range for all Enterobacter strains. Upon further investigation of aztreonam-avibactam resistant strains, whole genome sequencing was performed on the 7 *E. coli* isolates that had high aztreonam-avibactam MICs exceeding 8 μg/mL. In these isolates, 6/7 *E. coli* exhibited PBP3 (penicillin-binding protein 3) modification inserts, with 5 of these isolates also encoding an AmpC-variant (*bla*_CMY-42_) that manifested with increased activity against both ceftazidime and aztreonam. This unique finding was further investigated by Mushtaq, Livermore, and colleagues [29]. A representative sample of 464 MBL-producing Enterobacteriaceae was obtained from the UK Health Security Agency’s Antimicrobial Resistance and Healthcare Associated Infection Reference Unit. All Klebsiella and Enterobacter strains were susceptible in vitro to the combination of aztreonam-avibactam. However, only 85% of *E. coli* isolates (N = 122) were inhibited by this combination. Strains of NDM *E. coli* with a high aztreonam-avibactam MIC had a four amino acid insert in penicillin-binding protein-3 (PBP-3), often paired with an acquired AmpC β-lactamase. This finding is consistent with reports by Ma et al. [30] and those of Alm et al. [31], who described findings from 31 NDM-carbapenemase-producing Enterobacteriaceae. While all *K. pneumoniae* and *E. cloacae* isolates were inhibited by aztreonam-avibactam with an MIC ≤ 1 μg/mL, *E. coli* isolates exhibited a wide susceptibility range to aztreonam-avibactam with an MIC range of 0.125–16 μg/mL. Multisequence analysis of *E. coli* isolates with reduced aztreonam-avibactam sensitivity found that these strains had a four amino acid insertion after residue 333 in PBP3 [31]. This prompted an investigation for the presence of a similar 12 base-pair insertion in PBP3 within other Enterobacteriaceae species; however, within the sequences available in the public domain in GenBank, this insertion sequence was not seen in the 350 available *K. pneumoniae* or 75 *E. cloacae* sequences in GenBank. Conversely, this PBP3 insertion sequence variant was identified in 12 *E. coli* isolates from a total of 145 examined isolates; notably, none of which harbored an MBL β-lactamase. These isolates originated from India (7 isolates), China, Thailand, Turkey, Kuwait, and Lebanon [31]. This finding was reproduced in a study by Sadek et al. [32], whereas testing of 118 clinical *E. coli* MBL-producing isolates identified 16% with an aztreonam-avibactam MIC > 4 µg/mL and an additional 24% with a reduced susceptibility from 2–4 µg/mL. All isolates with elevated MIC had a four-amino-acid insertion within the PBP3 protein; however, this insertion sequence was also seen in susceptible strains; thus, while a contributor, it was deemed to be insufficient alone to confer resistance to an aztreonam-avibactam combination. Accordingly, all isolates with an MIC > 4 µg/mL were found to possess both a plasmid-borne *bla*_CMY-42_ gene, or rarely *bla*_CMY-2_, in addition to a PBP3 insertion sequence. Consequently, all isolates with MICs of aztreonam-avibactam of <0.5 µg/mL possessed a wild-type PBP3 sequence, regardless of the presence of any CMY-β-lactamase genes.

An additional concern relates to the potential impact of an inoculum effect on the in vitro activity of aztreonam-avibactam. Kim et al. [33] collected 81 clinical isolates of carbapenem-resistant *E. coli* or *K. pneumoniae,* of which 35 (43%) were carbapenemase-producers. Amongst this subgroup, while the aztreonam-avibactam in vitro sensitivity exceeded that of ceftazidime-avibactam (95% to 73%, respectively), an inoculum effect (defined as a ≥8-fold increase in MIC with a high inoculum of 10^7^ CFU/mL compared to a standard inoculum of 10^5^ CFU/mL) with aztreonam-avibactam occurred at a rate of 47%. An inoculum effect was substantially more common in aztreonam-avibactam than with ceftazidime-avibactam (18% occurrence) [33]. An additional notable finding was that the inoculum effect with aztreonam-avibactam occurred at a significantly higher rate in *K. pneumoniae* isolates (36/56; 64%) compared to *E. coli* (2/25, 8%). A unique finding reported by Li et al. compared the susceptibility of *K. pneumoniae* strains collected from clinical specimens at Sichuan Provincial People’s Hospital. Li and colleagues [34] screened 2529 *K. pneumoniae* strains and identified 154 carbapenem-resistance strains, of which 40 were identified as possessing a hypervirulent (hypermucoviscous) phenotype with a positive string test. Amongst these strains, the rate of aztreonam-avibactam in vitro sensitivity was 89.5% in the 114 carbapenem-resistant non-hypervirulent group, but only 75% in the 40 hypervirulent Klebsiella isolates. Interestingly, metallo-β-lactamase was not detected in any of the study’s hypervirulent strains, which predominantly harbored serine-based KPC-2 (83%). This raises concern that amongst Klebsiella isolates, reduced susceptibility to aztreonam-avibactam combinations may be facilitated by alternative mechanisms; specifically, Yu and colleagues [35] identified an association between resistant Klebsiella strains and high serine KPC (Ambler class A) expression in the presence of altered OMP35 and OMP37 (outer membrane porin). In contrast, Niu et al. [36] described excellent results in 68 MBL-producing *K. pneumoniae* isolates, including 13 that harbored dual carbapenemase (NDM and OXA) production, whereas the addition of avibactam reduced aztreonam MIC > 128-fold. Interestingly, genomic sequencing of mutant strains with in vitro selection of aztreonam-avibactam resistance identified mutations in the *bla*_CMY_ gene that reduced the inhibitory activity of avibactam in these CMY mutants. This led to a postulation that strains of Klebsiella with combined AmpC mutations, particularly those with additional outer membrane porin defects, could be a pathway for resistance to the novel aztreonam-avibactam combination. Within strains of *E. coli*, the presence of PBP4 variants harboring a truncated plasmid encoding a mutated *bla*_CMY_ AmpC β-lactamase has also been reported; these strains have reduced activity, as expected, to the aztreonam-avibactam combination [37]. A similar finding was reported in an experimental Enterobacter strain where a mutation in SHV-12 (Ambler class A) β-lactamase reduced the susceptibility to aztreonam-avibactam by significantly impairing avibactam inhibitory binding [38].

### 3.4. Clinical Experience with Aztreonam-Avibactam

Clinical experience with aztreonam-avibactam therapy remains limited. Yasmin et. al. [39] reported successful treatment in a 4-year-old child with hematologic malignancy and stem cell transplant with bacteremia due to an Enterobacter isolate harboring dual KPC and NDM MBL. Shaw et al. [40] described the use of ceftazidime-avibactam and aztreonam as salvage therapy during a hospital outbreak with *K. pneumoniae* producing multiple NDM-1, OXA-48, and CTX-M-15 β-lactamases. In ten patients, five were receiving immunosuppression therapy, and five patients had bacteremia at the time of initiation of combination antibiotic salvage therapy. Clinical success, survival, and lack of recurrence after 30 days were achieved in 6 of 10 patients. The combination of ceftazidime-avibactam with aztreonam has been successful in several cases, including the treatment of a case of MBL-*K. pneumonia* suppurative thrombophlebitis with persistent bacteremia [41], a case of NDM-1-producing *P. aeruginosa* pneumonia [41], a case of MBL-producing *Citrobacter sedlakii* tibial osteomyelitis [42], and a csase of NDM-1-producing *E. cloacae* prosthetic hip infection [43]. Benchetrit et al. [44] also described two cases of successful treatment of NDM-1 Klebsiella infection in organ transplant recipients, and Timist et. al. [45] reported microbiologic success in 9 patients in the intensive care unit that were afflicted with NDM-producing Enterobacteriaceae infections. The largest report describing the clinical use of aztreonam with ceftazidime-avibactam was by Falcone. Falcone and colleagues [46] performed a prospective observational study that enrolled 102 patients with bacteremia due to MBL Enterobacteriaceae, including 82 NDM-producers (79 *K. pneumoniae* and 3 *E. coli*) and 20 VIM-producers (14 *K. pneumoniae*, 5 *Enterobacter* spp., and 1 *Morganella morganii*). In this study, 52 patients received ceftazidime-avibactam and aztreonam, and 50 received best-alternative therapy, primarily combinational therapy including colistin, fosfomycin, tigecycline, meropenem, or gentamicin. The 30-day mortality in the aztreonam-ceftazidime-avibactam subgroup was 19.2%, compared to 44% in the comparison best alternative group. These findings were reinforced in propensity score-adjusted analysis, which found ceftazidime-avibactam with aztreonam had a hazard ratio of 0.37 with respect to 30-day mortality and a hazard ratio of 0.30 with respect to 14-day clinical failure rate. Mauri et al. [47] performed a systematic review including 35 in vitro and 18 in vivo studies on the combination of aztreonam-avibactam for MBL-producing Gram-negatives. In vitro data included 2209 isolates with susceptibility to aztreonam-avibactam in 80% of MBL Enterobacteriaceae, 85% of Stenotrophomonas, and 6% of MBL Pseudomonas strains. Clinical data were available for 94 patients, with resolution of infection in 80% of cases. Amongst patients (N = 64) with bloodstream infection due to an MBL Gram-negative pathogen, death occurred in 19% of patients treated with the combination of aztreonam and ceftazidime-avibactam.

Cornely et al. [48] reported results from a phase 2 REJUVENATE open-label multi-center study in patients with intra-abdominal infection. Patients received different dosing regimens of combination aztreonam-avibactam, with pharmacokinetic data measured. A loading dose of 500/167 mg administered over 30 min followed by a maintenance dose of 1500/500 mg via 3 h infusion every 6 h was determined to be the best option to achieve the target attainment goal of aztreonam exceeding an MIC of 8 mg/L for 60% of a dosing interval with a free concentration of avibactam exceeding a concentration of 2.5 mg/L for >50% of the dosing interval. However, in the absence of a current aztreonam-avibactam coformulation, clinicians rely on using the combination of intravenous aztreonam and intravenous ceftazidime-avibactam. This presents an additional challenge as an optimal dosing strategy has not been established. In vitro modeling by Lodise et al. [49] suggested that staggered administrations of ceftazidime-avibactam followed by aztreonam were less effective than simultaneous administration and that extended infusion (2 h infusion) enhanced killing compared to a 30 min infusion. Additionally, bactericidal activity was greater with 8 g/day aztreonam in experimental modeling compared to 6 g/day. Accordingly, Lodise [49] suggested an optimized dosing strategy of ceftazidime-avibactam 2/0.5 g every 8 h and aztreonam 2 g every 6 h. Subsequently, Falcone and colleagues [50] performed a prospective observational pharmacokinetic study in 41 critically ill patients (20% burn patients) who received treatment with combination ceftazidime-avibactam and aztreonam. Pharmacokinetic data were the basis for Monte Carlo simulation to develop a dosing nomogram. In these studies, aztreonam at 6 g/daily was sufficient to achieve the target attainment expected for an expected aztreonam MIC ≤ 4 μg/mL. Similarly, standard dosing recommendations for ceftazidime-avibactam exceeded the target threshold in all instances except when eGFR was between 6 and 15 mL/min, when increased dosing frequency (0.94 g every 12 h) was preferred to standard dosing (0.94 g every 24 h).

The COMBINE study was a phase I open-label study investigating the safety of ceftazidime-avibactam in combination with aztreonam in healthy adult volunteers [51]. Different dosing regimens were administered to six cohorts for a 7-day period. The experimental groups included a cohort administered 2.5 g ceftazidime-avibactam every 8 h via 2 h infusion in combination with 1.5 g aztreonam every 6 h (6-g group) via 2 h infusion, and a different cohort administered 2.5 g ceftazidime-avibactam every 8 h via 2 h infusion in combination with 2 g aztreonam every 6 h (8-g group) via 2 h infusion. The most frequently observed adverse event in the ceftazidime-avibactam with aztreonam cohort was hepatic aminotransferase elevation. This occurred in 50% (4 of 8) of the 6 g aztreonam group and 63% (5 of 8) of the 8 g aztreonam group. Elevations in AST/ALT were asymptomatic, resolved after cessation of drug, and were more commonly occurring in African American male subjects. Hepatic aminotransferase elevations were attributed to aztreonam administration, as the two highest elevations, ALT 600s and AST 400s, occurred in an experimental group that received only aztreonam 8 g via daily continuous infusion. Less frequently, mild grade 1 hematologic abnormalities (decreased hemoglobin, granulocytopenia, or thrombocytopenia) and mildly prolonged thrombin time were seen in experimental groups [51].

Das and colleagues [52] investigated dosing regimens for aztreonam-avibactam using population pharmacokinetic (PK) modeling and probability of target attainment analyses. Analysis attempted to determine optimal dosing to achieve > 90% target attainment with different dosing regimens with adjustment for patients with moderate and severe renal impairment. Avibactam was found to be the major limiting factor in achieving target attainment, with avibactam time free plasma concentration being the key parameter. Consequently, a 2.5-fold higher target level of avibactam was needed when used in combination with aztreonam compared to ceftazidime. The final iteration of PK modeling favored the use of an initial loading dose of aztreonam-avibactam 500/137 mg over 30 min. by maintenance with aztreonam-avibactam 1500 mg/500 mg every 6 h via extended infusion for creatinine clearance (CrCL) ≥ 50 mL/min. In patients with moderate renal impairment (CrCL > 30 to <50 mL/min), the recommended maintenance dose was 750/250 mg 3 h extended infusion every 6 h, and with severe renal impairment (CrCL > 15 to <30 mL/min), 675/225 mg 3 h extended infusion every 8 h [52].

Two additional phase III studies sponsored by Pfizer are currently investigating the combination of aztreonam-avibactam. The first trial, REVIST (NCT03329092), as a proof of concept, compared aztreonam-avibactam with metronidazole (if indicated) to meropenem with the optional addition of colistin in the treatment of Gram-negative bacterial infection. In the clinically evaluable cases, for intra-abdominal infection, the cure rate for the aztreonam-avibactam subgroup was 85.1% compared to the meropenem cure rate of 79.5%. In cases of healthcare associated pneumonia, the aztreonam-avibactam cure rate was 46.7%, in contrast to 54.5% in the meropenem comparator group. The 28-day mortality rate was low: 4/208 (1.9%) in the aztreonam-avibactam group compared to 3/104 (2.9%) in the meropenem group [53]. Unfortunately, one trial, ASSEMBLE, was prematurely terminated (NCT03580044) due to difficulty enrolling MBL infected patients. Prior to termination, 5/12 (41.7%) patients with confirmed MBL Gram-negative infections were cured with aztreonam-avibactam in comparison to 0/3 (0%) of patients that received best-available therapy [54].

## 4. Discussion

Current guidelines advise treatment of MBL CRE infection with one of two options, either the combination of ceftazidime-avibactam and simultaneous infusion of aztreonam or cefiderocol [5,45]. This is particularly notable when considering that cefiderocol, when combining data from the CREDIBLE-CR and APEKS-NP studies, achieved clinical cure of MBL Gram-negative bacterial infection in only 70.8% (17/24 cases); when focusing solely on MBL-producing Enterobacteriaceae, the clinical success rate was 73% (11/15 cases) [55]. A surveillance study by Kazmierczak et al. [56] found that while cefiderocol was active for 98% of 151 CRE isolates, susceptibility was only 58% amongst the 12 NDM-producing isolates. This finding is concordant with surveillance data that suggest NDM-producing Enterobacteriaceae have a higher MIC to cefiderocol than isolates that produce serine β-lactamases [5]. Current literature strongly supports high in vitro susceptibility to aztreonam-avibactam for MBL Enterobacteriaceae strains. This finding has been well documented and consistent across multiple geographic regions. In April 2024, the European Medicines Agency granted marketing authorization for Emblaveo, a single formulary combination of aztreonam-avibactam. However, at present, the combination of aztreonam-avibactam has not been FDA approved and is not available in the United States. Therefore, in clinical practice, the combination of ceftazidime-avibactam concurrently with aztreonam has been used, at least until a single formulary combination of aztreonam-avibactam becomes available.

While clinical experience remains relatively limited, initial studies have shown a high success rate in the treatment of MBL Enterobacteriaceae, which, at least in the study by Falcone and colleagues [46], significantly outperformed the best available alternative therapy. When considering the use of the combination of ceftazidime-avibactam and aztreonam, key limitations should be noted. Firstly, while aztreonam evades hydrolysis by MBLs, unlike other β-lactams that inhibit multiple PBPs, aztreonam has a potent but specific affinity for PBP3. This makes aztreonam vulnerable to mutational variance in PBP3 as compared to other β-lactams that exert activity against several different PBPs. Multiple studies have shown that PBP3 insert modifications are associated with resistance and failure of aztreonam-avibactam combinations; however, overwhelmingly, it appears that this occurrence is most predominant in *E. coli* strains. Additionally, *bla*_CMY_ AmpC gene mutations were found to reduce the inhibitory effect of avibactam and thus could be a precursor to developing resistance to the aztreonam-avibactam combination. However, the prior study by Sadek indicates that, absent PBP3 modification, the presence of *bla*CMY mutations was insufficient to independently result in aztreonam-avibactam resistance. Similarly, Klebsiella resistance was seen with hypervirulent mucoid strains, albeit with most of these cases harboring serine-based carbapenemase production. An additional limitation to the use of aztreonam-avibactam in clinical practice is the absence of readily available sensitivity testing of this combination. Therefore, as a priority in severe sepsis when the initiation of early effective antimicrobial therapy is critical, this limitation is particularly challenging.

**Figure 2:**
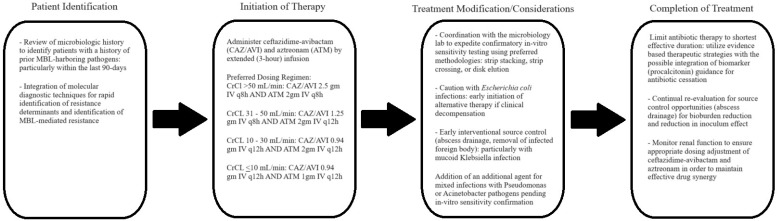
Diagnostic and therapeutic strategy for the selection of patients and use of ceftazidime-avibactam with aztreonam therapy. MBL = metallo-β-lactamase.

Based on the available current literature, a practical strategy for the use of aztreonam with ceftazidime-avibactam, recognizing the potential benefits and inherent limitations, can be devised (see Figure 2). As this regimen is primarily beneficial for MBL pathogens, integrating molecular diagnostic techniques to provide rapid identification of MBL-producing pathogens for early initiation is critical. Additionally, a careful evaluation of a patient’s historical cultures for prior isolation of MBL pathogens can also identify patients that have a high pre-test probability for MBL-producing Enterobacteriaceae infections, particularly if there has been a positive culture in the last 3 months due to the risk of persistent carriage [57]. While aztreonam with concurrent ceftazidime-avibactam has shown excellent activity in the majority of Enterobacteriaceae species, caution is needed with *E. coli* due to the potential for reduced activity—thus, it would be important to coordinate with the microbiology lab in order to set up early in vitro sensitivity confirmation testing, preferably with strip stacking, strip crossing, or disk elution methods as opposed to disk stacking. Additionally, lack of clinical improvement or inability to clear bacteremia should prompt early discontinuation and change to polymyxin, cefiderocol, or alternative therapy. Inoculum effects, particularly with hypermucoviscous Klebsiella strains, are also a risk for treatment failure, and thus, early interventions for bioburden reduction and source control, such as thrombectomy, abscess drainage, or surgical debridement, should be pursued. In the context of mixed infections with *Acinetobacter* or *Pseudomonas* spp., an additional antibiotic should be administered directed towards these pathogens to ensure effective active therapy pending in vitro sensitivity testing results. Based on available evidence, aztreonam at 2 g intravenous every 8 h (6 g/daily) with simultaneous administration of ceftazidime-avibactam is recommended; thus, multiple intravenous access catheters would be needed for optimal infusion. Additionally, administration of aztreonam by extended infusion (as opposed to a 30 min rapid infusion) would also maximally optimize bacterial killing. Continuous infusion does not appear to be required. The drug toxicity of dual ceftazidime-avibactam and aztreonam appears to be low, with a reversible asymptomatic aminotransferase elevation being the most commonly reported adverse effect. This effect was most pronounced in high-dose (8 g) aztreonam regimens, with a slight predisposition in African American patients. Therefore, liver function tests should be monitored, but absent clinical symptoms, aminotransferase elevation ≤ 5 times the upper limit of normal would be reasonably tolerated, especially in patients with severe sepsis. Administration of ceftazidime-avibactam at standard dosing appears sufficient for target drug level achievement at creatinine clearances > 30 mL/min; however, in critically ill patients, particularly in renal failure with an eGFR < 15 mL/min, increased dosing at 0.94 g every 12 h would be justified to ensure effective drug levels. Please see the recommended dosing strategies in Figure 2.

If in vitro sensitivity is confirmed, then treatment with ceftazidime-avibactam and aztreonam should be continued for the shortest effective duration to avoid selection of resistance. Utilizing biomarker-directed de-escalation, for example, procalcitonin, may be beneficial in this regard. Additionally, continual reassessment for optimizing source control procedures, particularly drainage of intra-abdominal abscesses (if present), is recommended. Monitoring renal recovery to ensure appropriate dose adjustment of both ceftazidime-avibactam and aztreonam will also be critical to ensuring effective drug synergy.

Ultimately, the option of ceftazidime-avibactam with aztreonam offers a valuable addition to the armamentarium for MBL Enterobacteriaceae infections; however, clear limitations prevent it from being a panacea. Novel agents, cefepime-taniborbactam and cefepime-zidebactam, were tested for 28 aztreonam-avibactam-resistant *E. coli* isolates. In these strains, unfortunately, 100% cross-resistance occurred with cefepime-taniborbactam; however, all strains were susceptible to cefepime-zidebactam [58]. Further investigation into the combination of aztreonam with novel inhibitor agents, zidebactam, nacubactam, and taniborbactam, has also shown promising results with MDR MBL-producing Enterobacteriaceae and *P. aeruginosa* clinical isolates and offers the potential for additional therapeutic options in the future [20].

## Data Availability

No new data were created or analyzed in this study.
